# Between Play and Exploitation: What Is the Place of the Rights of Child YouTubers?

**DOI:** 10.3390/ejihpe14050079

**Published:** 2024-05-01

**Authors:** Bárbara Morais Santiago Freitas, Natália Fernandes, Paula Gaudenzi, Bárbara Costa Andrada

**Affiliations:** 1 Instituto Nacional de Saúde da Mulher, da Criança e do Adolescente Fernandes Figueira (IFF), Fundação Oswaldo Cruz (Fiocruz), Rio de Janeiro 22250-020, Brazil; paula.gaudenzi@gmail.com; 2 Centro de Investigação em Estudos da Criança (CIEC), Instituto de Educação (IE), Universidade do Minho (Uminho), 4710-057 Braga, Portugal; natfs@ie.uminho.pt; 3 Núcleo de Pesquisas em Políticas Públicas de Saúde Mental (NUPPSAM), Instituto de Psiquiatria (IPUB), Universidade Federal do Rio de Janeiro (UFRJ), Rio de Janeiro 22290-140, Brazil; barbaracostaandrada@gmail.com

**Keywords:** children, rights, YouTube, play, exploitation, adults, YouTubers

## Abstract

This article aims to reflect on the images of childhood in videos featuring child YouTubers playing, analyzing the nature of play portrayed in them and its relationship with the child’s right to play and be protected against any form of exploitation. Method: A documentary study of 100 videos shared on YouTube was conducted, subjected to categorical content analysis with an emphasis on the modes of participation of adults and children in dialogues with the platform’s languages present in the videos. Results and Discussion: The boundaries between children’s artistic expression and child labor exploitation are becoming increasingly challenging, with legal discussions presenting difficulties in regulation due to the home environment and predominant parental control. The analysis reveals how the videos are perceived as standardized commodities, hiding the concrete work behind an image of apparent spontaneity. Conclusion: The research highlights contractual digital risks for children, focusing on those related to commercialization. The analyzed videos reflect an instrumentalization of the child’s basic right to play, associated with market interests, an aspect that takes on the contours of child labor exploitation, compromising the freedom to play spontaneously.

## 1. Introduction

The discussion presented in this article arises from a subset of a broader research proposal, specifically the dialogue of the ongoing research within the scope of the Doctoral Program in Public Health (IFF/Fiocruz, Brazil) by one of the authors, along with reflections stemming from her Scientific Doctoral Internship in Childhood Studies (IE/UMinho, Portugal).

The dataset for analysis was initially constructed to explore play in the context of digital media, focusing on videos shared on YouTube where children were described as “playing”. This resulted in an article that delves into a more detailed understanding of play in the analyzed videos [1]. However, upon a closer examination of these videos, we began to notice that the presence of adults was very prevalent, and the children’s participation seemed to stem from proposals made by adults. This led us to consider the possibility that these videos were not showcasing actual play but rather activities heavily directed by adults, with purposes extending beyond the playful dimension that children attribute to play. We observed a dimension of commodification in these videos, prompting us to question to what extent the child’s right to play might be manipulated in favor of other values, particularly the adults’ interest in financial gain through the apparent act of play by the child.

The topic of child YouTubers in Brazil has been under study. We have selected several studies, such as those by Tomé [2], which offer an in-depth analysis of Brazilian reality, Papini’s study [3] on unregulated child advertising on YouTube, and Melo and Guizzo’s [4] analysis of representations promoted by child YouTubers. Costa [5], in turn, questions whether the activity of child YouTubers constitutes child labor, highlighting the exploitation of child labor by the platform. Finally, one of the most relevant works in the field, by journalist and researcher Tomaz [6], reflects on how communicational processes affect childhood, pointing to new experiences provided by the production and consumption of digital content.

Our hypothesis is that the children we observed in the analyzed videos do not reflect merely spontaneous play but rather an activity conditioned by adults, with the end product being their “play”. We want to clarify that our intention is not to create an oppositional reflection between our hypothesis and the possibility of a playful dimension in this context, as we acknowledge that working children often find opportunities to play, even in extremely adverse situations [7]. However, we find it relevant to conduct a careful analysis of what is happening with these very young YouTuber children.

### 1.1. Thoughts about Play

We start from the understanding that play is complex and multifaceted, and the child’s universe, in which play is a fundamental element, is constructed based on the socio-economic context and utilizing collective, cultural, and historical–social values that shape play and representations of the world. We selected the works of psychoanalyst Donald Winnicott and philosopher Walter Benjamin in dialogue with the sociology of childhood as reading perspectives on play that guided our work.

According to Winnicott [8]: “to play is to make”; play and creativity go hand in hand in the subjective constitution and development of the child. Thus, when we talk about playing, we are talking about a significant action created by the child—an action that enters the symbolic world, in which the toy would be the material culture, an object that may or may not mediate the play. Through play, children elaborate their experiences, form their identity, and understand the world to which they belong. Benjamin [9] similarly emphasizes the creative and liberating aspects of play, suggesting that it should be experienced with time and dedication. For Benjamin, childhood is a social, historical, and cultural category, experienced by the child subject and not to be understood as a universal and solely biological category. There is an important difference between what we call playing and games. The games we refer to describe what was initially recognized as such in the analyzed collection, not necessarily leading to the symbolic and spontaneous dimensions that are essential for understanding play.

In the field of the sociology of childhood, there is an understanding that children constitute “a generational group with their own ways of interpreting the world, acting, thinking, and feeling, in which play assumes an unquestionable centrality, although it may adopt different facets depending on the latitudes in which it occurs” [10] (p. 13). Sarmento [11] proposes investigating the grammar of children’s cultures based on four pillars: interactivity, playfulness, the fantasy of reality, and reiteration. In this text, we highlight the axis of “playfulness”, which aligns with Winnicott’s view of the importance of play in mediating the external world (objects). From this perspective, play is a central element in understanding children’s cultures, even though playfulness is not exclusive to this generational category.

Since the mid-20th century, play has been included in the agenda of children’s rights, particularly with the recognition of its importance in the Declaration of the Rights of the Child [12]. In 1989, play was acknowledged as an autonomous right in the Convention on the Rights of the Child (CRC), especially in Article 31 [13]. In 2013, General Comment No. 17 published by the UN Committee on the Rights of the Child delved deeper into this theme. In Brazilian legislation, attention is drawn to references in Article 227 of the Brazilian Constitution [14], Article 16 of the Child and Adolescent Statute [15] and Article 17 of the Legal Framework for Early Childhood [16].

However, we are not solely interested in reducing this extensive debate to legal aspects. Tomás and Fernandes [10] caution against how play has been embraced by Western modern society as a “kind of guarantee of a healthy childhood and a central category in children’s lives worldwide, leading us to the idea of the (supposed) existence of a global model of childhood” (p. 16). Sarmento [17] defines cultures of childhood as “the ability of children to systematically construct ways of making meaning of the world and intentional action, which are distinct from adult modes of meaning and action” (p. 3–4). The intentional use of the term in the plural is emphasized by the author because one cannot overlook the fact that cultures of childhood are produced within societal cultures in which they are embedded, revealing intersections of social class, gender, race, country, etc. It is impossible, or reductionist, to speak of only one child culture, just as there is no single way of playing.

Understanding play as a central element in the construction of child cultures and recognizing that these cultures are not isolated from the larger cultures in which they exist, given the significant transformations witnessed in various societal domains, and considering the development of new information and communication technologies (ICTs), we are interested in thinking about play in the context of digital media.

### 1.2. Thoughts about Children in the Digital Environment

Research indicates a strong presence of the Internet and social networks in the daily lives of children, as seen in the Brazilian context, where 87% of children aged 9 to 10 and 96% between 11 and 12 use the Internet [18]. In Europe, based on EU Kids Online data, the majority of children reported using smartphones “several times a day” or “every day or almost every day” to access the Internet, with 84% of children and youth in Portugal expressing this behavior [19].

However, the change is not only in accessing the Internet but also in the possibility of producing and disseminating content. If, at the end of the 20th century, children only engaged as spectators of cartoons or children’s programs broadcast on television or in cinemas, now they can themselves produce videos, naturally linked to the accessibility of a smartphone and Internet connection. Indeed, the numbers have also increased in this context. In Brazil, 26% of children aged 9 and 10, and 32% aged 11 and 12, have posted their own text, image, or video [18]. This scenario illustrates how technology can also be used by children to publicize themselves. In light of the above, we can assume the significant role of technological mediation in shaping subjectivities for this age group, allowing more and more children to take on the role of digital influencers.

As we have seen so far, the digital environment has impacts at economic, cultural, social, and political levels in children’s lives. Based on this premise, thinking about and designing digital technologies that are safe and take into account the specificity of childhood is an important and urgent agenda in the field of children’s rights [20].

Regarding the international legal and ethical framework that governs children’s rights in the digital environment, we have as guiding norm the General Comment No. 25 published by the UN Committee on the Rights of the Child in 2021 [21], which was developed to address these issues. It emphasizes the need for both governmental bodies and digital companies to ensure that their actions and policies safeguard the best interests of children.

Hartung [22] presents the concept of Child Rights by Design (CRbD) to explore how to translate the principles and rights of the CRC into practice in the digital context. CRbD involves the design, development, and execution of online services or products used by children, defining specific measures for technology companies, designers, and developers in accordance with the best interests of children. One of the measures proposed by the author is the promotion of meaningful and non-monetizable experiences, described as “the design of the service or product should promote autonomous, playful and educational experiences, preventing the monetization of children’s experiences such as unauthorized artistic child labour” [22] (p. 7).

An essential contribution of our investigation is its reflection on potential risks faced by children in the digital environment. The latest and widely adopted categorization for addressing this issue in prominent Brazilian and European research is presented by Livingstone and Stoilova [23] as “the 4Cs classification”. This classification comprises content (exposure to potentially harmful content), contact (the risk of unsafe interactions initiated by adults), conduct (involvement in or victimization of harmful behaviors typically stemming from peer interaction), and contract (risks associated with commercialization and datafication). The researchers underscore the presence of cross-cutting risks, which manifest across all or most of the other risk categories.

We consider that the analyzed videos fall under contract risks, meaning they involve dynamics “where a child is party to and/or exploited by potentially harmful contract or commercial interests (gambling, exploitative or age-inappropriate marketing, etc.). This can be mediated by the automated (algorithmic) processing of data” [23] (p. 11).

Considering children as subjects entitled to human rights implies meeting a set of obligations and responses that safeguard childhood as a period for promoting their rights. The exploitation of children and their involvement in economic activities are deeply contradictory to this requirement.

### 1.3. Thoughts Child Labor in the Digital Environment

In the “Special protection measures” section of General Comment No. 25, the issue of child labor is specifically addressed:

Children should be protected from all forms of exploitation prejudicial to any aspects of their welfare in relation to the digital environment. Exploitation may occur in many forms, such as economic exploitation, including child labour, sexual exploitation and abuse, the sale, trafficking and abduction of children and the recruitment of children to participate in criminal activities, including forms of cybercrime. By creating and sharing content, children may be economic actors in the digital environment, which may result in their exploitation.

[21] (p. 19).

This passage discusses child digital influencers who achieve fame in the digital environment, especially on social media and platforms like YouTube, which are the subjects of our research, by producing content and profiting from it. In Brazil, this has the commonly misguided interpretation of not being considered artistic child labor. Consequently, it often evades judicial scrutiny, as required by child rights laws. This underscores the urgent need to debate ways to regulate this practice and ensure that it occurs in accordance with the best interests of children, including holding platforms accountable that encourage and profit from its monetization [24].

Jijón [25], in exploring definitions of child labor, highlights the heterogeneous ways in which children and their families characterize risks and motivations, and how the nature of the work itself can differ from what is postulated by the ILO (International Labour Organization). The relationship between children and adults is a central point in qualifying this experience. Jijón also emphasizes that children are active subjects and that even in work experiences, they may find spaces for play, not necessarily being entirely overshadowed by negative consequences.

Our interest here is not to characterize child artistic labor (CAL) precisely but to investigate how play, an activity marked by the spontaneity and uniqueness of the child, can be overlaid and permeated by consumer logics. We advocate for an analysis of work as a risk to biopsychosocial development and a violation of the rights of children involved in this context. However, it is important to situate what has been discussed regarding child artistic labor in the context of digital media, with a focus on discussions promoted by Brazil.

We begin with a brief overview of the legal situation initiated by the Convention No. 138/1973 of the ILO [26], which deals with the minimum age for admission to employment—set at 16 years and ratified by Brazil in 2002. However, Article 8 of this same Convention allows for an exceptional provision for participation in activities such as artistic performances for those under 16 years old. Authorization should be provided by competent public authorities, ensuring a series of conditions.

In the case of Brazil, we have Recommendation No. 139 [27], approved in 2022, which is a legal protective instrument reaffirming permits for the participation of children and adolescents in public performances. It emphasizes the need for the child or adolescent’s agreement, parental authorization and supervision, and compliance with the school routine. Resolution No. 245 [28], published in April 2024 by the National Secretariat for Children and Adolescents’ Rights of the Brazilian government, presents provisions aimed at protecting the rights of children and adolescents in the digital environment. By stipulating in Article 15 that “the personal data of children and adolescents should not be used for commercial purposes, such as creating and defining behavior, consumption, and market segmentation profiles” and by determining in Article 17 that “companies providing digital products and services used by children and adolescents (…) are responsible for implementing and ensuring the rights of this audience in the digital environments they produce and regulate”, the legislation assigns specific responsibilities to companies to ensure the protection of the rights of young users. These measures reflect an effort to promote the safety and well-being of children and adolescents online, addressing concerns such as the exploitation of child labor in the digital environment. The resolution is very new at the time of writing this text, and therefore, its effectiveness in dealing with these issues comprehensively cannot yet be determined. However, its promulgation underscores a growing concern for children’s rights in the digital environment, signaling a commitment to addressing and mitigating the challenges associated with this constantly evolving field.

In the United States, child actors are governed by the Coogan Laws, which ensure that part of their earnings is kept in a trust account until they reach a certain age—a strategy to curb parental abuse. Some initiatives seek to expand this legislation to digital environments [29], but as Masterson [30] points out, “child labor law inherently conflicts with family law and the constitutional protection of parental autonomy, particularly in the social media context. (…) Social media production is often overseen by the parents within their own home, and it can look like play, not work” (p. 580–581).

The discussion about explicit advertising in children’s videos that directly involve products and brands, such as controversial unboxing videos [31], where children unwrap toys and showcase them to the audience, is widely debated. It becomes evident that children are involved in marketing situations. However, when the product placement is less obvious, it becomes more challenging to characterize its marketing nature and potential harm, as observed in the videos we analyzed. Furthermore, many parents responsible for child-centered content deny that their children are working in this context, claiming it is merely play [32,33]. In our daily lives, we often hear many adults, especially parents and teachers, believe that because the videos show children playing, it is spontaneous content and, consequently, poses no risk to the children. 

There is a growing concern about defining boundaries about play and child labor: are they playful activities in which children play spontaneously? Or are they activities conditioned by adults, mimicking the act of play but, in reality, economic activities where the child’s labor is exploited under the guise of supposed play? [30,32,33,34,35]. About that, McGinnis says, “(…) lawmakers may find it increasingly difficult to distinguish children’s work online from play, as it often looks on camera like fun activities, unboxing, crafting, or creating—all while having fun with friends or family and under the supervision of parents or guardians” [32] (p. 260).

Therefore, considering (a) play as one of the central elements of children’s experience; (b) play videos as an important constituent part of contemporary play cultures; (c) the question of the manipulation of children’s right to play in favor of adult financial interests, our goal is to reflect on the images of childhood that emerge from the participation of very young child YouTubers in a set of analyzed videos. We aim to question the type of participation of children in these videos, the nature of play portrayed in them, and their relationship with the child’s right to play and be protected against any form of exploitation.

## 2. Materials and Methods

The video-sharing platform YouTube has proved to be very popular among children in various contexts. Research indicates that YouTube is the favorite site for children to access online videos [36,37]. Specifically in the European context, the platform has become increasingly popular among children [19]. Although we have noticed a growing use of other digital platforms by children, such as TikTok, a platform for sharing short videos [37], we have chosen YouTube for specific reasons. TikTok operates on a presentation screen that showcases content, which becomes increasingly personalized as the app is used [38]. This dynamic can make the search process complex and not always yield suitable results [39]. Given this context, we found that TikTok’s content, primarily tailored to individual preferences, would introduce significant methodological constraints for our purposes.

Understanding the significant role of peer culture in shaping children’s play culture, we chose to focus on videos featuring child protagonists. The concept of “peer culture” is invaluable to the sociology of childhood and can be broadly defined as “a stable set of activities or routines, artifacts, values, and concerns that children produce and share in interaction with each other” [40] (p. 158). While Corsaro [40] primarily refers to peer culture in terms of face-to-face interactions among children, it is worth noting that in today’s digital age, many children also engage (almost) daily with their peers through digital technologies. Thus, selecting videos with child protagonists allows us to tap into and explore this evolving digital peer culture, enriching our understanding of children’s play experiences.

Thus, we have defined the subjects that are the focus of our investigation: children featured in videos with content aimed at their peers. In Brazilian literature, these children are most commonly referred to as “youtubers mirins” (little YouTubers). However, we problematize this nomenclature, as we understand that it carries an adult-centric perspective, symbolically subordinating the child influencer to the adult. This observation was also made by Andrade [41] who, in their research, opted for the term “crianças youtubers” (YouTuber children), which we agree with. The relevance of content produced by Brazilian children extends beyond national borders; for example, there are records indicating that Portuguese children draw inspiration from videos produced by Brazilian YouTubers when recording videos, even if these are not published [42].

The main content of these channels, among the most viewed, includes daily life, travel, challenges, makeup tutorials, culinary recipes, and showcasing personal belongings such as toys and school materials [43]. Play-related videos (including traditional games, unboxing, challenges, pranks, and games) have been mentioned as the content that children most enjoy watching [2,4,5,44,45]. One of the most important studies on the subject, by Tomaz [6], observed that out of 123 analyzed videos, 100 were about play. Based on these findings, we focus on play-related videos shared on YouTube for our investigation.

We conducted a documentary study [46] using 100 videos shared online. The selection of videos began by entering the keyword “brincando“ (playing) into the YouTube search engine in April 2022. We want to clarify that the choice has significant implications on our dataset attributed to a linguistic nuance in Portuguese. The activities involving specific rules, such as those found in popular videos where children play online games like Roblox or Minecraft, are commonly referred to as “jogando” rather than “brincando” (both translated as playing). Our research focused on the concept of “brincando” (play), because we aimed to explore unstructured play devoid of external rules or objectives.

At the time of the research, the results were displayed in an “infinite scroll” style. Faced with the impossibility of knowing the total number of videos associated with our search, we decided to investigate how the digital field itself deals with these potentially infinite results. We found that sites like Google and others [47,48] typically highlight information around the first 100 results, and other studies [31,49] indicate that YouTube users usually do not view more than 100 results. Therefore, we concluded that the “top 100” strategy would be an interesting methodological approach, and we sought to construct the “top 100 videos of children playing”.

The search results are automatically made available according to “relevance”, but the specific criteria for this classification are not provided by the YouTube platform. Thus, we decided to use the “sort by” filter in the “view count” option, which displays videos with the most views. Our exclusion and inclusion criteria were carefully chosen to align with our research focus on analyzing playful cultures surrounding play, emphasizing the supposed spontaneity of play. We aimed to explore how these playful cultures manifest themselves through videos that are not professionally produced or have a declared professional character. In this manner, we watched the videos in the presented order and selected the first 100 that met the inclusion criteria, which were as follows: (i) play being the main theme of the video, (ii) the presence of at least one child directly involved in the play, and (iii) that this child was Brazilian. We excluded 57 videos based on the following reasons: 31 videos that did not feature children, 15 videos with foreign children, 7 animated cartoons, 3 music videos, and 1 episode of a TV series. This selection process was guided by our aim to focus on spontanoeus videos, not declared as professional children’s play videos.

**Figure 1:**
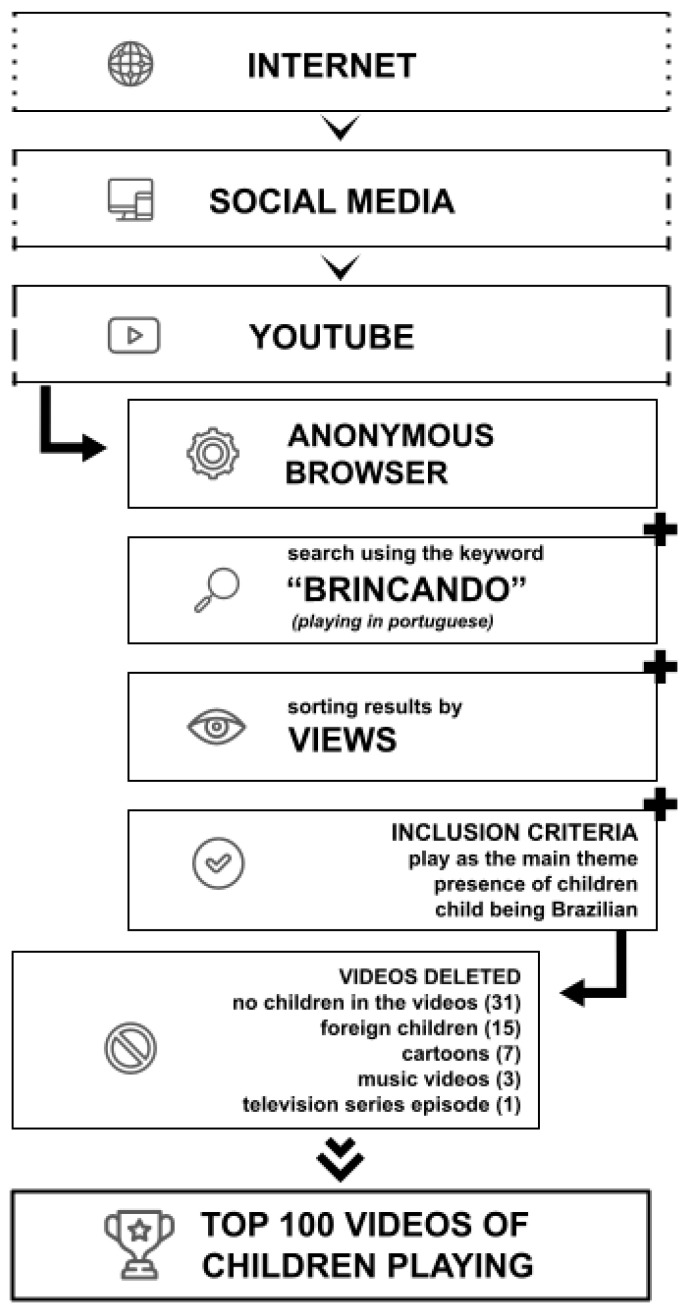
Methodology flowchart.

We collected information about the following aspects: video link, title, publication date, number of views, and how many times users interacted with the “like” and “dislike” buttons (Figure 1).

In a second phase, we re-watched the 100 videos that underwent a categorical content analysis [50]. We identified the “units of analysis” as the videos, considering audiovisual information, verbal and non-verbal languages, and the channels on which the videos were published. For this stage, we guided ourselves with the following research questions: What ways of play are shared through videos of children’s play on YouTube? What can these ways of play inform us about contemporary play cultures? We described, in the previously mentioned spreadsheet, the coding units divided into subgroups of analysis: characterization of the play, performance and characterization of the children, performance and characterization of the adults, and the platform’s languages. We want to highlight that we utilized emic categories because we aimed for the analysis categories to emerge directly from the field. Regarding the encoding process, it was initially conducted by the first author and subsequently validated by two other researchers, independently. For the presented analysis, we used findings from all subgroups, with emphasis on the last two (Table 1 and Table 2).

This research was not submitted to the Research Ethics Committee, following Brazilian recommendations and resolutions, as it is a documentary study of publicly and unrestrictedly accessible data [51,52]. However, aiming for an ethical stance and respecting the logic of protecting the privacy and confidentiality of YouTube information [53], we did not identify the children, adults, or channels that composed our sample.

## 3. Results

In total, 16 h and 54 min of videos were viewed. The videos had a minimum duration of 2:52 and a maximum of 1 h; most videos had a duration between 5 and 10 min (*n* = 54). The videos, as of April 2022, had between 7 million and 236 million views. Regarding the date of publication, the oldest were published in 2016 and the most recent in 2021. An important tool on YouTube is the “Like” and “Dislike” buttons. The user can select the icon of a hand with the thumb facing up for “Like” and another with the finger facing down for “Dislike”, located just below the video player. Regarding the amount of “likes” given to the video, we have 3 videos (from the same channel) that had more than 1 million, in 14 videos we found from 500 thousand to 1 million, 51 videos had between 100 and 500 thousand, and 31 had less than 100 thousand. The number of “dislikes” was unavailable in all videos, although it was possible to click on the button.

### 3.1. Mapping Adults Participation in the Videos

Adults are evidently present in 79% of the analyzed videos, totaling 112 individuals (52 men and 60 women). Of these, 96% are white, 1% are black individuals, and in 4 videos, the adult does not appear on camera. Regarding the relationship between the adult and the child, 80 adults were the parents of the children (60% mothers and 40% fathers), 19 were hired actors, just like the children; 5 were relatives such as uncles or grandparents, 4% were employees of the establishments the children were attending (teachers, recreational staff, etc.), and in 3%, it was not possible to establish the relationship.

Regarding the channels, 69% reference either individual children or a pair of siblings; 17% are family channels (parents and children, mother and daughter, larger family units, etc.); 10% are groups of actors that involve adults and children, and 3% consist of individual adults, but with a significant amount of content featuring children.

In 67% of the videos where adults appear, they are directly involved in the main play, either participating in games and challenges, acting out a role in dramatization, or exploring a space with the children. For example, video 21 begins by framing a mother and daughter. The 10-year-old child says, “*Hi, everyone, today I’m here with my mom*” to which the mother responds, *“Hi, everyone*”. They simultaneously say, “*Today we’re going to do the slime challenge*”. Then they announce the rules of the game: choosing colors while blindfolded. Both of them draw lots, mix the glitter glues, and show the results of each one’s slime. They conclude by saying, “*You* [pointing to the camera] *will decide who won the challenge!*”.

The adults, generally fathers or mothers, film while directing the child to perform a certain action, such as showing a toy better, interacting with the audience, without participating in the play in 32% of the videos.As we can see in video 35, the mother is filming two siblings without appearing in the scene. The 11-year-old girl announces that they are expecting a giant toy in the living room. The mother says, “[boy’s name], *when the man arrives with the toy, will you open the door?*” and the three discuss whether the toy will fit in the backyard. A team arrives and sets up the inflatable soccer field, inflating it in the backyard. The mother says, “*What will it be,* [girl’s channel name]?* What will it be,* [boy’s channel name]?” The 7-year-old boy enters the toy, and the mother says, “*This boy knows how to play! Throw the ball, son, throw!*”. The girl and the boy slide, jump, and score a goal with a ball. Then the girl is alone, and the mother says, “*Go ahead, honey, slide from there to here!*”.

In 22% of the videos analyzed, parents provide some support for the play “behind the scenes”, such as picking up objects, making comments about the place, etc. For example, in the video 61, two brothers play with unpacking a giant plastic excavator. They assemble the object and explore its functions. The father is filming, but even though he is not in the foreground, he participates in the play.

Finally, we observe some videos (5%) where adults lead the play; in this case, they are employees of certain establishments directing, but there are few videos with this characteristic. An example is video 20, where we see an 8-year-old girl arriving at a swimming lesson and meeting a friend. They change clothes and head to the pool. The girls swim following a series of instructions from the teacher, like “*Now you swim backstroke! Go!*”. Halfway through the video, she says, “*Now it is time for you to play however you want!*”. The girls point to a square foam, and the teacher gives some instructions. They finish the video with the teacher saying, “*Well done!*”.

Regarding the number of adults present in the scene, in 70% of the videos, only one adult appears; in 23%, there is interaction with two adults; and in 8%, there are more than three adults. In videos with more than one adult, it is observed that they have different roles or take turns in them. As we can see, video 50 begins with an adult dressed as a police officer, speaking to a 7-year-old girl also dressed as a police officer with a pink motorcycle, informing her that someone has escaped from prison. An adult dressed as a prisoner steals a doll from a 3-year-old child who then informs the two dressed as police officers. It is worth noting that the younger girl is dubbed by another person. The woman escapes in a car, and the girl police officer pursues her, retrieves the doll, and returns it. Based on the analysis of other videos from the channel, we affirm that it involves two parents and two sisters, all participating in the enactment taking place in the backyard of the house, with the parents taking turns filming.

We observed that 80% were filmed in residential environments (interior rooms, common areas of a condominium, backyard, etc.). For example, in video 10, we observe two boys aged 5 and 7, along with their parents, in one of the boys’ bedrooms. The video starts with one of the adults saying “*Folks, today we’re at* [boy’s name]*s’ house,* [son’s name]*s’ cousin, who’s going to show us his toys. Do you have a lot of toys?*”. The cousin responds by nodding affirmatively. Throughout the video, we witness several brief scenes of the children handling the objects and conversing with each other and the camera, with an adult eventually making an appearance.

Another dimension is how children react to adult demands. Let us take, for example, video 4, in which the father, mother, a girl, and a boy (2 and 6 years old, respectively) explore a toy store. The parents direct which toys the children should interact with, briefly showing various items on the shelves. The children, especially the boy, jump on a trampoline, drive an electric car around the store, shoot a basketball into a hoop, etc. In this relational context, one would expect that the children might want to buy a toy or spend more time on a particular activity, or even show no interest in something the adult finds interesting. However, none of these reactions are shown, and the children, even the very young girl, are only shown obediently complying with the adults’ requests.

### 3.2. Mapping Platform Language in the Videos

We can observe, especially in videos that are part of the same channel, the attempt to create catchphrases. We see the use of audience-related vocatives such as: “*Hi, YouTube folks*” or “*Bye, guys*” and in farewell videos with texts that are repeated literally, for example “*kisses on the heart, bye, bye and see you in the next video*” in video 26. Most of the videos appeal to the audience to interact with the platform’s tools, either by saying “*People, subscribe to the channel, press the bell and like!*”, or by using graphic animations with “subscribe” buttons” or “give a like”. Interaction with the audience is restricted to exploring these tools in almost all videos; some ask users if they are liking the content or voting through the comments (these are videos prior to the deactivation of comments on videos aimed at children).

In the coding unit “Interaction with the camera”, we mapped the following categories: interaction with users, talking directly to the camera, looking at the camera and showing an object to the camera. In all videos, at least one of these actions was observed in the children, demonstrating that they are aware that moment is being recorded and many of them respond to the “standard” behavior of videos on YouTube, indicating that they know that the content will be displayed on the platform. 

Video 3 is an interesting example of interaction with users and talking directly to the camera. The content produced consists of a 9-years-old-girl whose video’s theme is playing with “slime”; she announces the rules by speaking to the camera, even though she has two children beside her who participated in the play. The play consists of each child choosing items to make their slime and each one showing the result to the camera. The girl who “leads” the play always announces her actions by saying “*look, guys!*” addressed to whoever watches it. At the end she says “*See you in the next video, bye! It was really cool.* (…) *We’ll give you a little time to like, turn on notifications so you don’t miss out on all the fun*”.

In video 9, an 8-year-old girl plays pretend as the mother of a doll: she offers plastic food, changes its clothes, and performs actions like holding it and “giving a shot” to calm its crying. The play lasts almost 5 min, and the child looks at the camera while interacting with the doll 24 times, for example, when she says, “*Now that you’ve changed* [looks at the camera], *we can go to the pool!*”. In video 30, a 3-year-old boy is wearing a chef’s hat, sitting at a table, and explains that he’s going to play with a toy for melting chocolate. The father is filming and assisting. While handling the toy, the child says, “*Hey guys, do you know what’s inside here?* [showing a piece of the toy]”. The boy opens the object, revealing small red plastic spoons, and he shows each one to the camera.

In 67 videos, there is mention of another social network. This mention is made both in the children’s speech, in the description of the videos or in graphic insertions throughout, and at the end of the content. There is an interest in the statements that YouTube “followers” also follow children on other profiles. Instagram is the most cited social network, but we also note Facebook, TikTok, Musicall.ly, and Spotify, among others. In video 79, a 7-year-old girl and a 10-year-old boy conclude with the following alternating statement: “*Thank you very much to all of you who watched this video until the end, liked the video, and subscribed to the channel. And follow us on Instagram, which is appearing here below* [pointing to the names of two Instagram profiles individually referencing their names]”. This statement is repeated exactly with the same words in several videos of the channel, besides referencing other social media platforms in the channel description and video descriptions.

## 4. Discussion

In previous works [1], we have elaborated in greater detail on how we cannot immediately characterize the play exhibited in the videos. We have identified that the modes of play in the analyzed videos prominently highlight the paths paved by homogenization and commodification, ultimately leading to the monetization of play. In other words, within the analyzed videos, the creative play, as described by Winnicott [8] through the unrestricted use of symbols, is overshadowed by the unproductive repetition of meanings within the world. We conclude that these constitute what we term as “spectacle-play”. According to Hartung [22], the presence of child influencers on digital platforms such as YouTube can be considered an economic exploitation of the image and artistic data of these children when the following characteristics are present: external expectations, high frequency, and monetization.

Regarding the first characteristic of videos being influenced by external expectations, we observed that children do not utilize YouTube as a tool for self-expression or to share spontaneous content. Consequently, they are likely being guided by forces other than their own spontaneous play. We arrived at this conclusion because, in the top 100 most viewed play videos analyzed by us, we observed passive responses to adult demands, scripted behavior, and a homogenization of content.

The dynamic, where adults direct play and children do not throw tantrums or refuse to participate in anything, readily engaging in activities, is what we observed throughout our dataset. In the analyzed videos, children respond promptly to the suggestions and directions of adults, showing no behavioral resistance to the guidance. We hypothesize that if any child displayed oppositional behavior, it might have been subject to editing so that it does not appear in the videos. This presents an image of an idealized child who readily follows adult instructions and parents who are always available for play, without any relational noise. This makes us ponder how these play scenarios are not spontaneous or, if they were, underwent some form of editing to construct a narrative external to the original context.

In many videos, we encounter play motivated by the objects on the scene, clearly predefined actions that tell us little about the child’s subjectivity. This is play confined to a predetermined script. Furthermore, we consistently observe children incorporating scripted lines into their performances, particularly about the features of a product (brands, functions, etc.), raising questions about the spontaneity of what is presented in such content. For instance, in video 16, featuring a father and son interacting with building blocks—a globally recognized brand with a virtual gaming theme—the boy presents the box, shows the instructions, and assembles it exactly as indicated. We see brief play that reproduces the narrative of the themed game.

Another aspect that leads us to question the spontaneity of what is shown in the analyzed videos is the post-production elements, such as editing, visual graphics, sound effects, and musical scores. We notice a significant homogenization of these resources, which, in another analysis, we concluded to be part of the YouTube genre of content for children [1].Similar findings were found by Nicoll and Nansen [31] when analyzing unboxing videos: “Young and amateur YouTubers possess an awareness of the affective and technical literacies that inform the production of successful toy unboxing videos” (p. 11). The authors point out that there is a two-way influence, as more professional channels try to reproduce an atmosphere of authenticity and playfulness by appealing to a more amateur aesthetic, while smaller channels replicate the communication strategies of those established adult content creators on the platform.

Even though these videos may seem spontaneous and unscripted, the reality is that many are meticulously planned to attract views, expand the follower base, and ultimately generate revenue through sponsorships, partnerships, and advertising. In other words, as highlighted by Guzman [29], it is crucial to acknowledge that the parents’ decision to film, edit, and publish videos of their children on YouTube involves financial profit.

According to Tomé [2], videos produced by child YouTubers depict an idealized childhood, where it is impossible to determine whether what is seen on the screens is spontaneous or performative and has been influenced by “strategic uses of the platform to expand the audience and the use of advanced features, demonstrating characteristics of professionalization” (p. 114). Based on both the young age of the children involved and the interest in adapting the content to the platform’s language, we believe that these interventions are carried out by adults, reflecting their interests rather than the creative potential of the child.

However, our findings suggest that what is displayed in these videos, described as children playing, may not reflect play but instead present a standardized commodity, concealing the concrete work behind a spontaneous image: “The collaborative culture that prints the idea of collaborators and partners further disseminates this ideology that deceives with the gimmick that the more you collaborate, the more the company grows, and if it grows, it grows equally.” [5] (p. 71). According to Frazão [54], “the increasingly massive use of the Internet by children is also the result of a marketing strategy by the platform” (p. 40). The entanglement between the content of the analyzed videos and YouTube’s market strategy can be perceived in various dimensions.

The second characteristic presented as characteristic of CAL is the high frequency of content production, not only in the sharing of videos but also with the aim of keeping the audience engaged. To gain insight into these numbers, we try to understand how often children were involved in videos published monthly analyzing our dataset. On average, each content creator posts four videos per month. However, the larger the channel (meaning the more subscribers it has), the higher the frequency of video uploads, with some channels posting daily. The most impactful example was the following: a channel which features a mother and her daughter, with over 2 million subscribers, posted 40 videos in December 2017. All these videos featured the child’s participation, and at the time, she was five years old.

Even though adults may handle the editing and channel management, this increased frequency of postings indicates that children spend a significant amount of time involved in video production. Other investigations also point out that child YouTubers experience a recording routine and financial expectations that resemble the adult world, involving obligations and concerns typical of an adult work routine [3].

Another aspect arising from the analysis is that, generally, these children are active on other social media platforms such as Instagram, TikTok, and Facebook. Additionally, we find an ecosystem of channels in which they participate (for example, family channels, collaborations on other children’s channels, etc.), leading us to assert that these children’s dedication to online content production is intense and not restricted to either the YouTube platform or the main channel. We have four channels from our analysis corpus that are not the children’s main channels but self-identify as “Family [child’s name]”. One of them has the following description: “*Welcome to the Family* [child’s name] *channel! Here you will have a lot of fun with videos of little stories, tales, games, and much more. Be part of this big family! Email: management@*[child’s name].*com.br*” and had almost 4 million subscribers, while the main channel, which only referenced the child’s name, had 11 million subscribers.

The possibility of video production characterizing a situation of child artistic labor is greater in “large YouTube channels”, as previous research has pointed out [4,55]. The investigated channels often “started as a playful endeavor” in an unpretentious manner. However, as the number of subscribers and views increased, routines and equipment took on the forms of labor, with children dedicating many hours of their day to video production. According to Costa [5], having a successful channel, as analyzed by her, does imply a form of child labor.

As Kuehl [33] emphasizes, “It is not play if you’re making money off it”. YouTube is a company with its own agenda, which often does not prioritize the best interests of children. Various forms of monetization are observed, including traditional advertisements and the sale of channel-branded products. According to Masterson [30], a prevalent argument among parents of influencer children is that they are merely capturing the child’s routine activities; in other words, they are not working, they are just playing. However, the researcher argues that this stance appears weak considering the substantial frequency of appearances by these children in numerous social media content and the active role of parents in filming their children for online audiences.

The first observation is that all the videos in our analysis corpus featured advertisements throughout their display. In other words, as we watched the videos, advertisements for some product or service were presented, both in the traditional format, where videos produced by the advertising company appear with variable durations ranging from seconds to over 10 min, and small frames displayed over the watched video. The content of such ads varies widely, not necessarily targeting the child audience, and changes with each view. We understand that advertisers are not specifically linked to a particular video and are displayed based on the platform’s algorithm parameters.

The display of ads only occurs if the channel owner chooses to enroll in the YouTube Partner Program (YPP) and thus participate in the ad revenue displayed in the videos [56]. When the user applies to join the YPP, there is an explicit interest in monetizing video sharing on the platform. Therefore, we have content that is clearly influenced by the interests of visibility and income generation, an aspect that does not seem compatible with the spontaneity that children’s play should embody, considering the risk that, to achieve higher view numbers, content may be shaped to respond to what will bring more returns.

We observed mentions of events and products related to the channels’ “brand”. They are presented both at the end of the video with a traditional advertisement, as well as in the video in which a kitchen routine is carried out and at the end we find the girl’s speech with illustrative images saying “*Embark with me on this adventure in the* [title of the book with the name of the child]! *You can find the book on the* [bookstore chain] *website and in all stores in Brazil! The link to buy is in the video description. Kisses!*” or inserted during the narrative, as in the case of a video whose plot revolves around a doll inspired by one of the children and which bears the name of the channel. In addition to books and dolls, we find collections of clothing and decorative objects. Regarding the events, book signing sessions and meetings with followers were mentioned. Although they do not appear very frequently in videos, we know that YouTube is not the preferred space for promoting these activities.

As an aggravating factor, child influencers do not have clear rights to ensure the management of their earnings or safe working conditions, putting them at a higher risk of exploitation [30].

It is noteworthy that the participation of adults in children’s channels occurs in a way that is not always verifiable in the videos. McGinnis [32] highlights the common practice among parents of circumventing minimum age restrictions on social media platforms by including disclaimers in their child’s account bios, asserting parental responsibility for the account’s creation and maintenance. In fact, the guardians or parents of the child are somehow present from the creation of the channel, as inferred from the messages many channels place in their descriptions, similar to this: “*In accordance with YouTube’s terms and conditions, this channel is owned and managed by* [child’s name]*s’ parents*”.

We believe this aligns with a policy initiated by YouTube in 2019 regarding access control for children under 13. This change was triggered by a $170 million fine imposed by the United States Federal Trade Commission (FTC) for privacy violations of minors [57]. Currently, the platform allows children to have accounts, but they are not authorized to have their own channels [58]. Thus, even if the child presents themselves as the content creator of the channel, the account’s responsible party must be over 18. 

Moreover, it can be assumed that many adults exclusively dedicate themselves to content production, especially in the case of large channels, as indicated by data from other studies such as Tomaz’s [6] ethnography with child YouTubers and in news reports on the subject [33].

Furthermore, as described earlier, the adults who appear in interactions with the children and take a more active role in content production are mostly family members, primarily mothers and fathers. Thus, we conclude that in the analyzed videos, parents are the figures evidently leading the video productions, constituting an activity that takes place in the domestic environment.The key factor to consider in regulating and safeguarding children in this context is the parental autonomy have in making decisions regarding the use of their child’s image on social media [32]. The challenge for the regulation of activities performed by child YouTubers lies in being an activity mediated by parents and conducted at home [30,32].

## 5. Conclusions

Upon analyzing the videos, we observe a lack of significant actions originating from the child’s inherent qualities such as their childhood cultures, playfulness, and spontaneity. Instead, the videos predominantly showcase scenarios where the adult assumes the primary role in proposing, guiding, and manipulating. Beyond this observation, another dimension becomes apparent, prompting us to introduce a commodification aspect into the analysis. In our view, this aspect distances or even contradicts the child’s right to play, potentially representing a denial of this fundamental right.

Based on that, it is crucial to emphasize the importance of characterizing that even videos labeled as play can be interpreted as forms of child labor, given the dynamics observed in our analysis. While parents may argue that their children are simply “having fun”, the fact that these children are involved in a wide range of social media content and that parents are actively filming their children for online audiences suggests exploitation issues. This characterization is essential to recognize and properly address the ethical and legal issues surrounding child labor on digital platforms, ensuring the well-being and rights of children youtubers.

Exploiting a fundamental child right, like the right to play, by linking it to market interests that require ongoing content production for financial gains represents, from our perspective, a contemporary manifestation of child exploitation. This approach strips away from the child the freedom to play in a spontaneous, uninhibited manner guided by their own desires and transforms it into a task-like activity constrained by rules and the economic interests of adults.

In conclusion, it is important to emphasize the need to generate inputs for the discussion about child artistic labor in the digital environment. Our work aimed to contribute to this discussion by underscore the complexity of the scenario where a child is seemingly playing. This distinction is crucial to ensure that the rights and well-being of children are adequately protected.

One limitation lies in the difficulty of extrapolating the data to other socio-cultural realities, due the videos analyzed being only about Brazilian children. Another one concerns the dynamic nature of online content, with fluctuating view counts and content nature, which may quickly render our analyzed data outdated due to the volatile nature of the online world. Additionally, an important methodological consideration pertains to the selection of the search term “brincando” (playing), which may have excluded videos of children engaged in play labeled with other terms.

## Figures and Tables

**Table 1:** Encoding—Adults’ participation.

Coding Unit	Description
Presence in the video	Identify if adults were explicitly present in the videos (Categories: yes and no)
Interaction with play	Record how the adult(s) participated in the central play. (Ex: participate directly, film, direct, etc.)
Child’s reaction	Describe the child’s reactions to the interaction initiated by the adult (Ex: resistance, passivity, etc.)
Relationship with the child	Classify the relationship with the child (Ex: mother, father, relative, employees, etc.)
Characteristics	Describe when possible gender and race/ethnicity.

**Table 2:** Encoding-Platform language.

Coding Unit	Description
YouTube-specific language	Identify the use of catchphrases, interaction with viewers, references to platform features
Interaction with the “camera”	Record if the child interacts with the camera
Other social media	Make references to other social media platforms
Events or products	Identify if there is advertising of own products or promotion of events related to their name

## Data Availability

We are unable to provide the acquired data as it would compromise our commitment to safeguarding the privacy and confidentiality of the individuals involved. Disclosing this information would contravene our principle of refraining from identifying the children youtubers, as outlined in Brazilian recommendations and resolutions, as well as in YouTube’s privacy policies.
